# Perception-action coupling in anticipation research: a classification and its application to racket sports

**DOI:** 10.3389/fpsyg.2024.1396873

**Published:** 2024-07-23

**Authors:** Kim Huesmann, Florian Loffing

**Affiliations:** ^1^Department of Sport and Movement Science, Institute of Sport Science, Carl von Ossietzky University of Oldenburg, Oldenburg, Germany; ^2^Section Performance Psychology, Institute of Psychology, German Sport University Cologne, Cologne, Germany

**Keywords:** ecological dynamics, embodied cognition, sensorimotor expertise, experimental design, representativeness, interaction, in-situ

## Abstract

Anticipation is key to performance in many sports. By definition, anticipation as a perceptual-cognitive process is meant to inform action and help athletes reduce potential motor costs under spatiotemporal pressure. Anticipation research has repeatedly been criticized for neglecting action and raised the need for predominant testing under conditions of perception-action coupling (PAC). To the best of our knowledge, however, there is a lack of explicit criteria to characterize and define PAC conditions. This can lead to blurred terminology and may complicate interpretation and comparability of PAC conditions and results across studies. Here, we make a first proposal for a 7-level classification of PAC conditions with the defining dimensions of stimulus presentation and response mode. We hope this classification may constitute a helpful orientation for study planning and reporting in research on anticipation. Further, we illustrate the potential utilization of the PAC classification as a template for experimental protocol analysis in a review on anticipation in racket sports. Analysis of *N* = 115 studies reported in *N* = 91 articles confirms an underrepresentation of representative PAC conditions and reveals little change in PAC approaches over more than 40 years of research in that domain. We discuss potential reasons for these findings, the benefits of adopting the proposed PAC classification and reiterate the call for more action in anticipation research.

## 1 Introduction

In many sporting contexts, athletes’ performance depends on their skill to accurately foresee what is likely to happen in the next moment. In the sports science literature, that skill is traditionally referred to as anticipation (Abernethy, 1987; Loffing and Cañal-Bruland, 2017).^1^ Specifically, anticipation is understood as the perceptual-cognitive process of predicting near future events (e.g., an opposing tennis player’s type and direction of serve) to enable optimal temporal and spatial alignment of one’s own actions with these events (e.g., return of serve). Hence, anticipation is meant to guide an athlete’s action. In view of this working definition of anticipation, scientists in the field frequently point out that research on anticipation needs representative testing environments that preserve the naturally occurring coupling between perception and action to draw conclusions on anticipation in the “real world” situation targeted in the investigation. More than 15 years back, for example, a focused debate emerged on the need for perception-action coupling (PAC) in sport-related anticipation research in a special issue of the *International Journal of Sport Psychology*. In their target article, van der Kamp et al. (2008) adopted an ecological approach and argued that in order to depict how athletes anticipate actions on field, perception and action need to be linked in a representative way. Similarly, Crognier and Féry (2007) conducted a review on anticipation research in tennis and criticized the broad use of experimental methods that separate the natural coupling of perception and action (also see Dicks et al., 2009). Until then, experimental approaches that evoke comparatively artificial PAC such as verbal or button responses to video stimuli in the lab were vastly employed ‘by tradition’ in sport-related anticipation research (Farrow and Abernethy, 2003). These approaches may come with certain benefits, such as better controllability of the testing environment, tasks, manipulation of independent and measurement of dependent variables as well as potential confounders which ultimately strengthen an experiment’s internal validity. Additionally, feasibility of data collection in terms of cost, space and time efficiency might lead to a tendency to favorite comparatively artificial over more representative experimental designs. However, the experimental design of a study and its associated degree of representative^2^ PAC influence the conclusions for research and practice scientists can infer toward real-world human behavior (Maselli et al., 2023; Raab et al., 2023). In this regard, the dominance of experimental approaches with little representative PAC in combination with an inconsistent description of PAC conditions potentially limits or even misleads our understanding of the sensorimotor processes underlying anticipation during sport-specific action and the expert advantage associated with it (for critical discussion, e.g., see Abernethy et al., 1993; van der Kamp et al., 2008; Dicks et al., 2009; Navia et al., 2018; Araújo et al., 2019).

Theories from ecological psychology propose a close bidirectional link between perception and action during movement execution. According to the ecological dynamics perspective (Araújo et al., 2006), there is continuous, ever evolving interaction between the environment, the actor and the task. This interaction produces affordances, that is opportunities for action, an actor can perceive and act upon. Experienced, in comparison to less experienced performers, are more attuned to relevant information and thereof evolving functional affordances which ultimately results in expertise-differences in anticipation and decision-making. However, the perspective argues that for real world (expertise in) anticipation and decision-making, actors need the possibility to perceive and act upon representative affordances (Travassos et al., 2017). The theories find support in sport scientific research investigating the influence of different PAC degrees on anticipation which indicate differences in anticipation and expertise effects depending on the experimental design and consequent PAC degrees used in studies (Farrow and Abernethy, 2003 in tennis; Ranganathan and Carlton, 2007 in baseball; Mann et al., 2010 in cricket; Huesmann et al., 2022 in handball). Beyond the ecological approach, other theoretical accounts also assume a close connection between perception and action, however, often with specific focus on particular aspects such as action observation, planning, initiation and execution (for an overview and systematization, e.g., see Gentsch et al., 2016).

Irrespective of the theoretical lens a study is specifically motivated by, the balanced discussion of results obtained from anticipation research and their associated potential implications (e.g., for practitioners) requires that researchers are able to describe and readers are able to understand, among others, the degree of PAC realized in a study. Two intertwined issues arise in this regard. First, to the best of our knowledge, there is a lack of explicit criteria to characterize and define PAC conditions. Second, the terms used for labeling the conditions under which participants are required to anticipate are frequently used inconsistently across studies. For example, Farrow and Abernethy (2003) asked participants to return a tennis serve in-situ and differentiated between two PAC conditions (uncoupled: verbal response; coupled: hit successful return stroke). Shim et al. (2005, Exp. 1), in turn, asked their participants to perform time-coupled on-court actions in response to tennis ground strokes presented either as point-light display, normal video (life-size screen projection) or a real opponent. The authors referred to all experimental conditions as perception-action coupled tasks without differentiating based on stimulus presentation. As another example, Ranganathan and Carlton (2007) used a virtual batting environment to study the effect of PAC by comparing batters’ performance when giving an uncoupled verbal response as opposed to swinging a baseball bat against the virtual ball. The latter condition was considered coupled although realistic ball interception was not required. Collectively, without criticizing previous works’ empirical merit, the former examples illustrate that different methodologies may underly the same label of PAC, whereas similar methodologies may underly different labels of PAC. Altogether, this potentially complicates interpretation and comparability of PAC conditions and results across studies.

Here we aim to take a first step toward a standardized PAC classification for anticipation research. Specifically, in the following section we propose a set of criteria to characterize different response modes in combination with different types of stimulus presentation for PAC classification. We then use this classification to exemplarily review the PAC conditions applied in research on anticipation in racket sports as a follow-up and extension to the overview provided by Crognier and Féry (2007) to illustrate the prevalence and temporal evolution of PAC approaches in that particular domain.

## 2 Perception-action coupling classification

Different levels of PAC have previously been defined based on the variation of participants’ *response mode* (Farrow and Abernethy, 2003; Farrow et al., 2005; Mann et al., 2010). We agree that this is the primary dimension along which PAC levels should be differentiated. However, we suggest that *stimulus presentation* should be added as a second dimension to allow more fine-grained differentiation of PAC levels (cf. Table 2 in Crognier and Féry, 2007, for a similar way to differentiate experimental protocols in anticipation research). This specifically applies to methodological approaches requiring a realistic (i.e., sport-specific), full-body movement either in response to videos or virtual reality as opposed to an in-situ condition against a real opponent. In the former situation, full coupling of a motor response to the stimulus display is not possible due to the missing opportunity for real interception, whereas in the latter case, interception is possible and participants may be specifically instructed to act accordingly (Farrow and Abernethy, 2003; Shim et al., 2005; Dicks et al., 2010). Further, consideration of stimulus presentation helps reduce the sole impact of response mode. We argue that requiring participants to act does not constitute a stronger case for PAC *per se* compared to when participants are not required to act but to, for example, make a verbal response. Specifically, this applies to situations when participants are confronted with still images as these do not provide a continuous flow of visual information participants’ action might be coupled with (in contrast to, e.g., video or in-situ).

**Figure 1:**
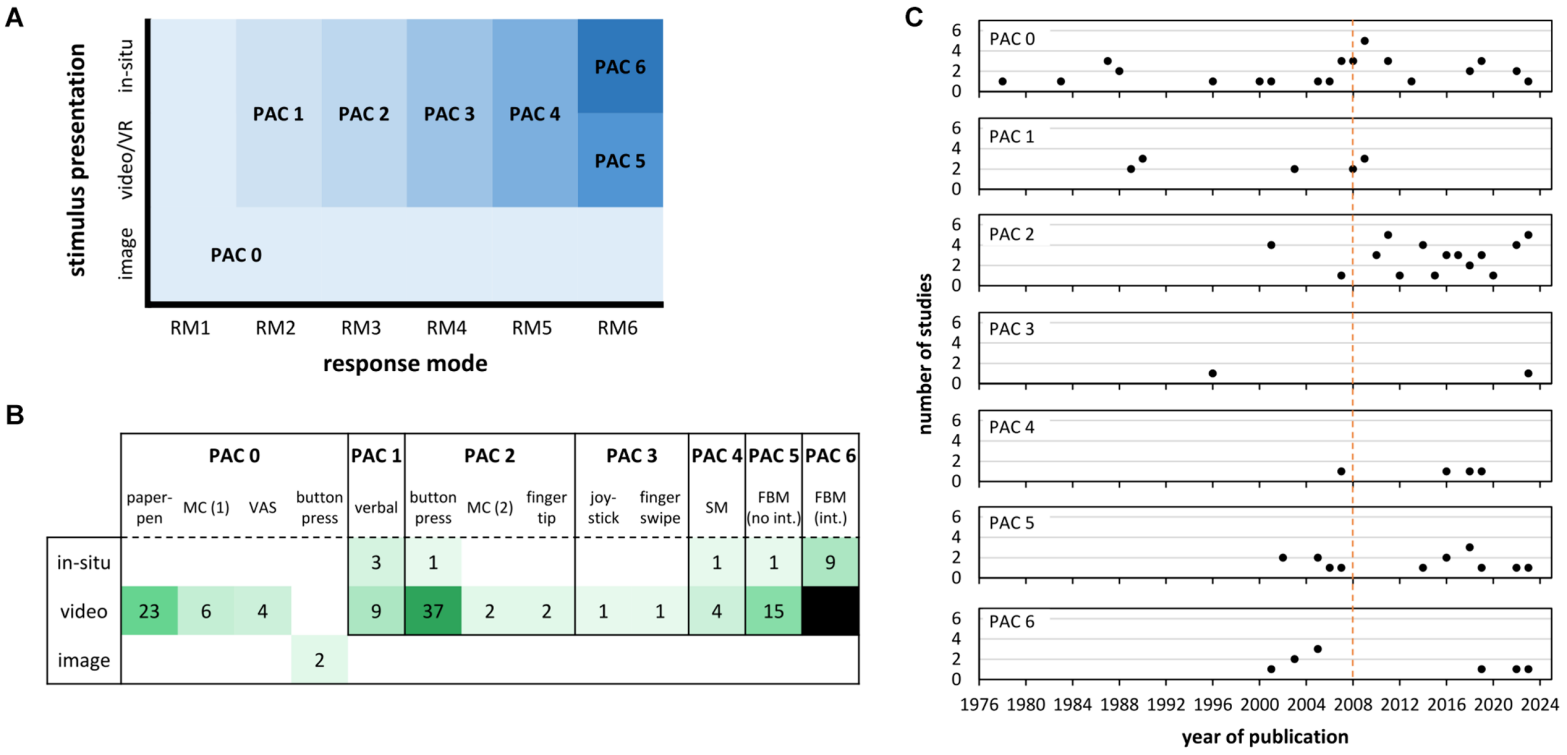
**(A)** Levels of perception-action coupling as defined by response modes RM1-6 and stimulus presentation (for details see Table 1). **(B)** Number of studies on anticipation in racket sports using a particular combination of response mode and stimulus presentation. Combinations are assigned to different levels of perception-action coupling (PAC) according to the criteria listed in Table 1. **(C)** Number of studies assigned to each PAC level by year of publication. The vertical dotted line indicates the year of a special issue on the coupling of perception and action in anticipation research with the much-regarded target article by van der Kamp et al. (2008). MC (1) = mouse click on a court representation (digital analog to a paper-pen response); MC (2) = mouse click (left/right) as directional response similar to button press; VAS = visual analog scale; SM = simple movement (e.g., foot toward left/right); FBM (no int.) = full-body movement (no interception); FBM (int.) = full-body movement with interception; PAC = perception-action coupling.

The classification we propose differentiates between seven levels of PAC (see Figure 1A; Table 1). The lowest (PAC 0) and highest level (PAC 6) indicate conditions of no and full representative PAC, respectively. For the first PAC-defining dimension of *response mode*, we suggest to differentiate between six modes as outlined in Table 1. Each of these modes is defined by five characteristics related to a response’s temporal proximity to stimulus (far/near), its spatial alignment with stimulus (no/yes), temporal resolution (discrete/continuous), device used (artificial/realistic), and task-specificity of the action (non-specific/specific). Characteristics were chosen to enable unique assignment of response modes and to reflect a response’s increasing representativeness (with regard to the constraints within the testing environment) relative to real-life demands from lowest (RM1) to highest mode (RM6). Examples for each of the six response modes are given in Table 1. For the second PAC-defining dimension of *stimulus presentation*, we suggest to differentiate between three types: still image, video/virtual reality (VR) and in-situ (see Travassos et al., 2013, for an identical differentiation of stimulus presentation). These types differ according to the flow of optical information related to an opponent’s action (image: none; video/VR & in-situ: continuous) and the perceptual richness the to-be-anticipated action is embedded in (image to in-situ: very low to high).

**Table 1:** Response mode and stimulus presentation as the two defining dimensions for the levels of perception-action coupling.

	**Response mode**						**Stimulus presentation**			
Code	(1) Temporal proximity to stimulus	(2) Spatial alignment with stimulus	(3) Temporal resolution	(4) Device	(5) Task-specificity	Example	Image	Video/VR	in-situ	Dependent variables, e.g.
RM1	Far	No	Discrete	Artificial	Non-specific	Paper-pen	0			Accuracy/error, response time
RM2	Near*	No	Discrete	Artificial	Non-specific	Verbal	0	1		Accuracy/error, response time
RM3	Near*	Yes	Discrete	Artificial	Non-specific	Button press (left, right)		2		Accuracy/error, response time
RM4	Near	Yes	Continuous	Artificial	Non-specific	Joystick, mouse		3		Same as above + movement time, movement dynamics (e.g., trajectory, corrections)
RM5	Near	Yes	Continuous	Realistic	Non-specific	Simple movement, e.g., step left/right		4		Same as above
RM6	Near	Yes	Continuous	Realistic	Specific	Full-body movement, e.g., tennis return		5	6 (only if interception possible)	Same as above + temporal/spatial error relative to estimated position of projectile, sport-specific skill execution (e.g., timing, velocity, angle), temporal/spatial error, response outcome (e.g., serve return performance)
							PAC levels			

* Temporal proximity for discrete responses contingent on task instructions, here assuming that participants are required to act timely as if in real competition. PAC, perception-action coupling; VR, virtual reality.

We suggest to define PAC levels based on a combination of response mode and type of stimulus presentation. As still images do not provide a continuous flow of optical information toward which a response could be aligned irrespective of mode, we classified this as PAC 0. Also, responses of mode RM1 are classified as PAC 0 irrespective of stimulus presentation because this mode offers the lowest opportunity for representative action and its coupling with perception irrespective of the stimulus (see Table 1 for details on the characteristics of RM1). Specifically, requiring participants to respond, e.g., via paper-pen means they need to change attention from the stimulus display (e.g., video, image) to the response display (e.g., sheet of paper), thus inducing a temporal gap between stimulus and response (i.e., far temporal proximity, see Table 1).

PAC levels 1–4 are defined solely based on response modes RM2 to RM5 and apply similarly to video/VR and in-situ within respective response modes (Table 1; Figure 1A). The guiding principle in differentiating these PAC levels is the gradual change along the five response mode characteristics. Specifically, at PAC 1 temporal proximity is near (e.g., verbal response can be given temporally connected to a critical stimulus event), but a response’s spatial alignment with a stimulus is still not possible. The latter, however, is possible from PAC 2 onwards (e.g., left button press when a participant anticipates their opponent to hit a ball to their left). Further, at PAC 2, a response is still discrete and in PAC 3 responses are still given using an artificial device (e.g., via joystick) but they are continuous. Continuous responses allow for spatiotemporal corrections in the course of movements and thus may inform about changes of mind due to, e.g., updated beliefs regarding the most probable target for action (Savelsbergh et al., 2002; Cos et al., 2021). At PAC 4, the response device becomes realistic (i.e., the body) but the required response is still task-unspecific (e.g., simple movement such as step left/right). Overall, changes from far to near temporal proximity (PAC 0 ➔ PAC 1), possibility for spatial alignment with stimulus (PAC 1 ➔ PAC 2), from discrete to continuous responses (PAC 2 ➔ PAC 3) and from an artificial to a realistic device (PAC 3 ➔ PAC 4) create increasingly representative affordances, that is opportunities for action, which allow a step-by-step approximation to real-world task demands.

Finally, PAC 5 and 6 are characterized by task-specific responses (e.g., full-body movement such as tennis serve return). These two levels are further differentiated by *stimulus presentation*, with PAC 5 and PAC 6 applying to video/VR and in-situ conditions, respectively. Research realizing designs at PAC 5 and PAC 6 levels may inform about how anticipation guides action, the dynamics underlying cue utilization throughout the full course of an evolving opponent’s action as well as the spatiotemporal adequacy of the full movement response. If a study is conducted in-situ and a task-specific full-body movement response without interception is required, we recommend to classify that condition as PAC 5. We suggest that a condition should be classified as PAC 6 only if participants are instructed to perform a realistic interception, for instance, of a ball moving toward them in the real-world performance environment (e.g., on-court in tennis). Consequently, the key difference between PAC 5 and PAC 6 is that PAC 6 might additionally allow for the assessment of response outcome (e.g., serve return performance; Table 1). This is not or only restrictedly (e.g., in VR) possible at PAC 5 and lower levels. Thus, in our view the assumption that anticipation is meant to guide an athlete’s action is most extensively reflected in experimental protocols using PAC 6.

The PAC classification is meant to be used as orientation for the development of experimental protocols at the stage of study planning and the description of methods in, for instance, manuscripts to prepare a targeted discussion of results. Moreover, the classification may also be used as a template to analyze the experimental protocols reported in the literature. We next report the exemplar application of the PAC classification to cross-sectional research on anticipation in racket sports. By doing so, we aim to illustrate its use and reveal the prevalence and the temporal evolution of PAC approaches as a basis for a critical discussion of methodological developments and recommendations for future work.

## 3 Perception-action coupling approaches in racket sports

### 3.1 Methods

We followed the updated PRISMA guidelines (Page et al., 2021) and systematically searched the scientific literature using the search term [anticipat* AND (tennis OR “table tennis” OR badminton OR squash OR padel)] in three databases (PubMed [search field: all fields; *n* = 167], Web of Science [Core Collection; search field: topic; *n* = 341], Scopus [search field: title-abs-key; *n* = 279]; last updated search on February 13, 2024).^3^ Original, peer-reviewed articles that reported a cross-sectional approach to investigate anticipation in racket sports were included if the full text was published in English or German. No restriction was made neither on the year of publication nor on the research discipline (e.g., sport science, psychology, neuroscience). Original studies reporting a training intervention were excluded just like any forms of reviews, meta-analyses, conference proceedings, project reports, book chapters or articles published in languages other than English or German. Moreover, studies were only included if sport-specific stimuli were presented (e.g., video opponent in a study on tennis serve return), and were excluded if a sport-*un*specific type of stimulus (e.g., runway of LEDs to investigate coincidence-anticipation; Ripoll and Latiri, 1997; Le Runigo et al., 2005) or only a ball machine was implemented (e.g., Alain et al., 1986) as both do not provide participants the opportunity to anticipate based on, for instance, advance kinematic (e.g., opponent’s shoulder rotation) or contextual (e.g., opponent’s on-court position) information. Also, studies were excluded if no measure of anticipation but only participants’ visual search behavior was recorded on-court (e.g., Lin et al., 2021; Espino Palma et al., 2023). Finally, studies that were of purely observational nature were excluded because of the lack of at least partial control of the testing environment, although in general we consider such work highly valuable and informative with regard to describing anticipatory behavior in real racket-sport competition (e.g., Howarth et al., 1984; Triolet et al., 2013; Mecheri et al., 2019; Benguigui et al., 2024). The search protocol as well as an overview of the included studies, publication years, sports and experimental methods can be found in the Supplementary material. The focus of descriptive analysis using Microsoft Excel was on identifying the type of stimulus presentation and response mode as basis for determining the PAC level realized in the included studies.

### 3.2 Results

A total of *N* = 91 articles published between 1978 and 2023 were identified eligible for inclusion (see Supplementary Figure S1 for a PRISMA flowchart). Most articles reported studies in tennis (*n* = 60), followed by badminton (*n* = 18), table tennis (*n* = 10) and squash (*n* = 3; none in padel). Overall, the articles reported a total of *N* = 115 studies (due to several multi-study reports) that were considered for a methodological analysis of PAC approaches (see Supplementary Table S1 for an overview of individual studies’ classifications).

Accordingly, as is illustrated in Figure 1B, in most studies, participants viewed videos and responded via button press, a combination that belongs to PAC 2 according to our classification (see Table 1). The second most employed combination again was to show videos and ask for paper-pen responses (belonging to PAC 0), followed by full-body movements in response to videos (belonging to PAC 5), verbal responses to videos (belonging to PAC 1) and full-body movements with in-situ interception (belonging to PAC 6). When the different response mode and stimulus presentation combinations were considered overall and assigned to PAC levels, PAC 2 was most often realized, followed by PAC 0, 5, 1, 6, and 3 (Figure 1B).

A differentiated view on the number of studies conducted per PAC level by the year of publication is given in Figure 1C. Accordingly, research realizing the lowest level of PAC has the longest tradition and this approach is used regularly until today (PAC 0; top row in Figure 1C). Research realizing a PAC 2 (e.g., button press in response to video), in turn, has gained popularity from 2010 onwards. Requiring participants to perform full-body movements in response to video (PAC 5) or in-situ with interception (PAC 6) was introduced in the early 2000s (e.g., Abernethy et al., 2001). However, these methodological approaches did not become the dominant ones from then on, especially not in comparison to PAC 2 (Figure 1C).

## 4 Discussion

Different experimental designs all carry a particular degree of PAC. Motivated by theories that assume a close link between perception and action, such as the ecological dynamics perspective, researchers have repeatedly criticized the dominant separation of perception and action in studies on anticipation (Abernethy et al., 1993; Araújo et al., 2006, 2019; van der Kamp et al., 2008; Travassos et al., 2013). Consequently, there has been unequivocal call for more sport-specific action and the preservation of representative PAC to further our understanding of expert athletes’ exceptional sensorimotor skills on the field (Prinz, 1997; van der Kamp et al., 2008; Gentsch et al., 2016; Maselli et al., 2023). Additionally, the current inconsistent use of terminology for the description of experimental methods caused by a lack of explicit criteria to characterize and define PAC conditions renders the proper interpretation of (the scope) of available studies difficult.

Here we made a first proposal for a criteria-based classification of PAC levels. The classification is hoped to help standardize methodological terminology, enable more targeted discussion of the results’ scope and to stimulate the systematic, theory-driven comparison of the impact of different PAC levels particularly on anticipation, but potentially also on related perceptual-cognitive skills (e.g., decision-making, pattern recognition). Additionally, the classification is neutral in the sense that it is applicable to different sports and tasks (e.g., batting in cricket and baseball, goalkeeping in soccer and handball). As exemplified in our application to anticipation research in racket sports, the PAC classification may also serve as a template for the analysis of experimental protocols reported in the literature to identify methodological foci and trends as well as to reveal potential methodological gaps worth addressing and experimentally challenge theoretical predictions.

Our review revealed persistent and predominant use of rather artificial PAC approaches that especially require discrete button press or paper-pen responses (i.e., PAC 0-2; see Figures 1B,C). Thus, little seems to have changed over more than 40 years of anticipation research or since the much-noticed call for more action by van der Kamp et al. (2008; see the number of studies relative to the dotted orange line in Figure 1C). This, at least, pertains to the domain of racket sports and includes our own research on anticipation (e.g., Loffing et al., 2016; Huesmann and Loffing, 2019). However, we expect that the methodological landscape would not noticeably change when extending the view on other interceptive sports and tasks (for methodological advancements in research on decision-making, e.g., see Inns et al., 2023; Iskra et al., 2024).

Despite the evident calls for more action in anticipation research, why did higher PAC levels not become more common and maybe even standard over time? We suspect that the underlying reasons are manifold and intertwined. For example, experiments with low PAC are less resource demanding (e.g., button press in response to videos shown on a notebook monitor, results stored in easy to process logfiles) than experiments with high PAC (e.g., large labs or gyms and real opponents required, recording of participants’ full-body movements, potentially in combination with motion capturing, as response mode, enhanced complexity in data processing and analyses due to multivariate datasets). Considering the research questions targetable with the different PAC levels, however, studies with lower PAC should not be discarded as less valuable in comparison to high PAC studies. Low-PAC studies might allow for a targeted and resource-efficient initial investigation of, for instance, the influence of selected information sources on skilled anticipation and this might then be followed up by studies using higher PAC to, e.g., test transfer to the field. Finally, as another potential reason, the evidence from studies that investigated the influence of different degrees of PAC on anticipation to date is indicative but not overly convincing (Mann et al., 2007; Travassos et al., 2013), for example, with regard to a more pronounced expertise advantage (Ranganathan and Carlton, 2007; Mann et al., 2010) or better anticipation performance at higher than lower PAC levels (Farrow and Abernethy, 2003; Huesmann et al., 2022). The classification presented here may help as orientation for future systematic theory-driven analysis of such PAC effects.

Still, we emphasize the call for strengthening the action component in anticipation research and to purposefully use it as an (in)dependent variable to, for instance, experimentally challenge the proposition that perception and action are linked bidirectionally (Araújo et al., 2006; Lepora and Pezzulo, 2015; Pizzera, 2016; Maselli et al., 2023; Voigt et al., 2023). Emerging technologies, such as for instance VR, can help researchers create experimental designs with high degrees of PAC while still allowing close control of the experimental setting, combining the advantages of high and low PAC levels. Further, there is continued interest in answering the questions on which kinematic and contextual sources are used for and how related information is computationally weighted and integrated in skilled anticipation (Loffing and Cañal-Bruland, 2017). The recent literature argues in favor of Bayesian computational models (Harris et al., 2022; Gredin et al., 2023), however, part of the evidence supporting this idea originates from approaches with rather low PAC using paper-pen (e.g., Harris et al., 2023) or button press (e.g., Loffing and Hagemann, 2014; Helm et al., 2020; for exceptions, see, e.g., Gredin et al., 2018; Murphy et al., 2018; Magnaguagno and Hossner, 2020). In our view, preserving the representative coupling between perception and action in this line of research (e.g., see Mann et al., 2013; Stone et al., 2014, for enabling realistic ball interception under controlled stimulus conditions) would make an even stronger case for ‘Bayesian anticipation’ and facilitate transfer of gained insights to the real setting where such computations are assumed to guide skilled performance.

Finally, the PAC classification proposed here (Figure 1; Table 1) is not without limitations. For example, we did not consider and further differentiate PAC levels depending on whether the temporal or spatial occlusion technique is part of an experimental protocol. This may be relevant, however, to keep in mind for, among others, in-situ studies that use liquid crystal googles to occlude participants’ vision before ball flight. In situations like these, realistic task-specific interception will be difficult to achieve (e.g., Farrow and Abernethy, 2003). Also, PAC levels were defined without specifically differentiating between task instructions such as whether participants are required to respond as fast (or timely) and accurately as possible within a specific time frame or without time constraints. Instead, we implicitly assumed that the first type of instruction is used but if not, we suggest to report so and even consider downgrading a particular PAC level because the response’s temporal proximity to a stimulus might not be given.

Overall, we would like to reiterate that the classification is meant as a first step toward a criteria-based systematization of PAC levels, but it is explicitly not meant as a tool to assess the quality of experimental methods used in studies. In that sense, we hope the classification will constitute a helpful orientation for researchers that facilitates both study planning and reporting, that it can serve as a tool for experimental protocol analysis as well as aid the transfer of knowledge to practice by classifying the methodological level of PAC at which evidence was obtained. Beyond the application to racket sports shown here as an example, the PAC classification can also be applied to other interceptive sports and tasks. Based on our historical record of the PAC levels realized in anticipation research in racket sports, we expect a similar pattern of findings in other domains of sport and would like to express the anticipatory wish of “A little more action, please!.”

## Data availability statement

The original contributions presented in the study are included in the article/Supplementary material, further inquiries can be directed to the corresponding author.

## Author contributions

KH: Conceptualization, Formal analysis, Methodology, Visualization, Writing – original draft, Writing – review & editing. FL: Conceptualization, Formal analysis, Methodology, Supervision, Visualization, Writing – original draft, Writing – review & editing.

## Footnotes

^1^Occasionally, the term *visual anticipation* is used in the literature to indicate the prominent role of vision for anticipation (e.g., van der Kamp et al., 2008). We have deliberately omitted this specification as, in addition to visual information, auditory information, for example, presumably influences the anticipation process (e.g., Cañal-Bruland et al., 2018). Moreover, anticipation also relies on memory processes (e.g., knowledge of the opponent’s action tendencies, general event probabilities; Loffing and Cañal-Bruland, 2017), so that we consider the neutral term anticipation more appropriate here.

^2^The term “representative” is defined by the ideas of Brunswik (1956), who stated that representative study design represents the typical environment and task constraints of the “real world task” targeted and intended to be generalized upon in the investigation (for an application of Brunswick’s ideas to sports science see Pinder et al., 2011).

^3^Note that our review was not aimed at providing a full systematic review in the sense of an (almost) exhaustive coverage of the literature but rather to showcase the application of the PAC classification. Therefore, we kept the PRISMA route of identification of studies via other methods rather short and consequently cannot rule out that we have missed some reports, especially from earlier years, that are often not listed in databases (Supplementary Figure S1).
