# Supplementary material for: Perception-action coupling in anticipation research: a classification and its application to racket sports
Source: Front Psychol. 2024 Jul 23;15:1396873. doi: 10.3389/fpsyg.2024.1396873 (PMC11300321; doi:10.3389/fpsyg.2024.1396873)
Supplement: Supplementary file 1 [file Data_Sheet_1.pdf]

Supplementary Material

Supplementary Figure 1. PRISMA flowchart for the review on (quasi-)experimental cross-sectional studies on anticipation in racket sports (see main text for details). Note that the approach was not meant to provide a full systematic review in the sense of an (almost) exhaustive coverage/overview of the literature but rather to showcase application of PAC classification (see main text for details). Therefore, we kept the PRISMA route of identification of studies via other methods rather short and consequently cannot rule out that we may have missed some reports, especially from earlier years, that are often not listed in databases.

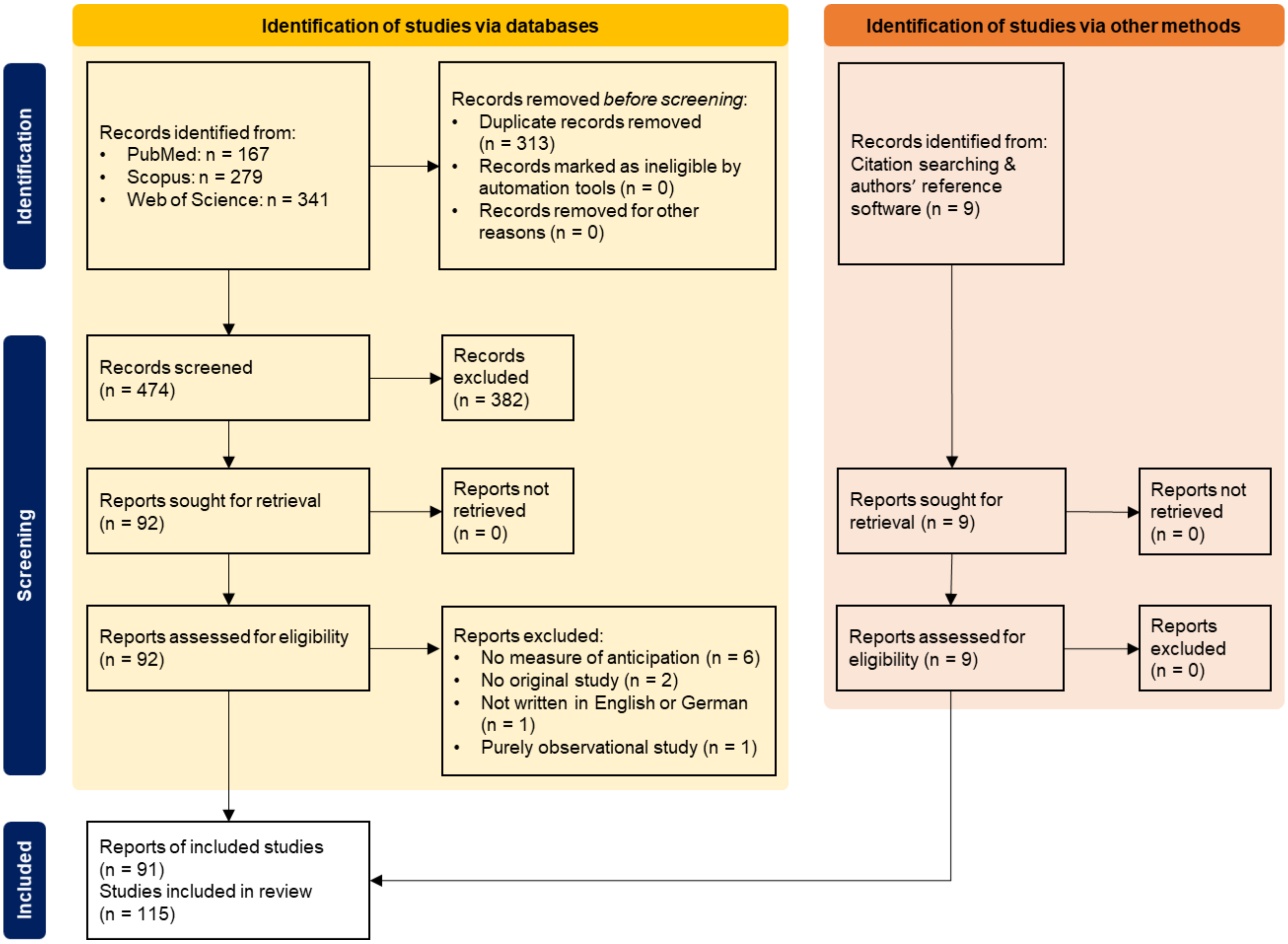

Supplementary Table 1. Overview of reviewed studies on anticipation in racket sports upon which the PAC classification was applied (see main text for details).

no	retrieved via	reference	study	year	sport	stimulus presentation	response mode	level of perception-action coupling							note
								0	*1*	*2*	*3*	*4*	*5*	*6*	
1	other method	Jones, C. M., & Miles, T. R. (1978). Use of advance cues in predicting the flight of a lawn tennis ball. Journal of Human Movement Studies, 4, 231-235.	---	1978	tennis	video	paper-pen	x							
2	other method	Isaacs, L. D., & Finch, A. E. (1983). Anticipatory timing of beginning and intermediate tennis players. Perceptual and Motor Skills, 57(2), 451-454. https://doi.org/10.2466/pms.1983.57.2.451	---	1983	tennis	video	paper-pen	x							
3	other method	Abernethy, B., & Russell, D. G. (1987). Expert-novice differences in an applied selective attention task. Journal of Sport Psychology, 9(4), 326-345. https://doi.org/10.1123/jsp.9.4.326	1	1987	badminton	video	paper-pen	x							
4	other method	Abernethy, B., & Russell, D. G. (1987). Expert-novice differences in an applied selective attention task. Journal of Sport Psychology, 9(4), 326-345. https://doi.org/10.1123/jsp.9.4.326	2	1987	badminton	video	paper-pen	x							
5	database	Abernethy, B., & Russell, D. G. (1987). The relationship between expertise and visual search strategy in a racquet sport. Human Movement Science, 6(4), 283-319. https://doi.org/10.1016/0167-9457(87)90001-7	---	1987	badminton	video	paper-pen	x							
6	database	Abernethy, B. (1988). The effects of age and expertise upon perceptual skill development in a racquet sport. Research Quarterly for Exercise and Sport, 59(3), 210-221. https://doi.org/10.1080/02701367.1988.10605506	---	1988	badminton	video	paper-pen	x							
7	other method	Buckolz, E., Prapavesis, H., & Fairs, J. (1988). Advance cues and their use in predicting tennis passing shots. Canadian Journal of Sport Sciences, 13(1), 20-30.	---	1988	tennis	video	paper-pen	x							
8	other method	Goulet, C., Bard, C., & Fleury, M. (1989). Expertise differences in preparing to return a tennis serve: A visual information processing approach. Journal of Sport & Exercise Psychology, 11(4), 382-398. https://doi.org/10.1123/jsep.11.4.382	1	1989	tennis	video	verbal		x						
9	other method	Goulet, C., Bard, C., & Fleury, M. (1989). Expertise differences in preparing to return a tennis serve: A visual information processing approach. Journal of	2	1989	tennis	video	verbal		x						

no	retrieved via	reference	study	year	sport	stimulus presentation	response mode	level of perception-action coupling							note
								0	*1*	*2*	*3*	*4*	*5*	*6*	
		Sport & Exercise Psychology, 11(4), 382-398. https://doi.org/10.1123/jsep.11.4.382													
10	database	Abernethy, B. (1990). Anticipation in squash: Differences in advance cue utilization between expert and novice players. Journal of Sports Sciences, 8(1), 17-34. https://doi.org/10.1080/02640419008732128	---	1990	squash	video	verbal		x						
11	other method	Abernethy, B. (1990). Expertise, visual search, and information pick-up in squash. Perception, 19(1), 63-77. https://doi.org/10.1068/p190063	1	1990	squash	video	verbal		x						
12	other method	Abernethy, B. (1990). Expertise, visual search, and information pick-up in squash. Perception, 19(1), 63-77. https://doi.org/10.1068/p190063	2	1990	squash	in-situ	verbal		x						
13	database	Singer, R. N., Cauraugh, J. H., Chen, D., Steinberg, G. M., & Frehlich, S. G. (1996). Visual search, anticipation, and reactive comparisons between highly-skilled and beginning tennis players. Journal of Applied Sport Psychology, 8, 9-26.	---	1996	tennis	video	button press & joystick				x				joystick response (for ball flight direction) was taken to determine PAC level; button press was required for type of serve response
14	database	Tenenbaum, G., Levy-Kolker, N., Sade, S., Liebermann, D. G., & Lidor, R. (1996). Anticipation and confidence of decisions related to skilled performance. International Journal of Sport Psychology, 27(3), 293-307.	---	1996	tennis	video	paper-pen	x							
15	database	Tenenbaum, G., Sar-El, T., & Bar-Eli, M. (2000). Anticipation of ball location in low and high-skill performers: A developmental perspective. Psychology of Sport and Exercise, 1(2), 117-128.	---	2000	tennis	video	paper-pen	x							
16	other method	Abernethy, B., Gill, D. P., Parks, S. L., & Packer, S. T. (2001). Expertise and the perception of kinematic and situational probability information. Perception, 30(2), 233-252. https://doi.org/10.1068/p2872	1	2001	squash	video	paper-pen	x							
17	other method	Abernethy, B., Gill, D. P., Parks, S. L., & Packer, S. T. (2001). Expertise and the perception of kinematic and situational probability information. Perception, 30(2), 233-252. https://doi.org/10.1068/p2872	2	2001	squash	in-situ	full-body movement							x	with ball interception
18	database	Féry, Y. A., & Crognier, L. (2001). On the tactical significance of game situations in anticipating ball trajectories in tennis. Research Quarterly for Exercise and Sport, 72(2), 143-149. https://doi.org/10.1080/02701367.2001.10608944	---	2001	tennis	video & in-situ	button press			x					for both conditions - in-situ & video - PAC level coded as "2" because button press required in both conditions
19	database	Rowe, R. M., & McKenna, F. P. (2001). Skilled anticipation in real-world tasks: Measurement of attentional demands in the domain of tennis. Journal of Experimental Psychology: Applied, 7(1), 60-67.	1	2001	tennis	video	button press			x					

no	retrieved via	reference	study	year	sport	stimulus presentation	response mode	level of perception-action coupling							note
								0	*1*	*2*	*3*	*4*	*5*	*6*	
20	database	Rowe, R. M., & McKenna, F. P. (2001). Skilled anticipation in real-world tasks: Measurement of attentional demands in the domain of tennis. Journal of Experimental Psychology: Applied, 7(1), 60-67.	2	2001	tennis	video	button press			x					
21	database	Rowe, R. M., & McKenna, F. P. (2001). Skilled anticipation in real-world tasks: Measurement of attentional demands in the domain of tennis. Journal of Experimental Psychology: Applied, 7(1), 60-67.	3	2001	tennis	video	button press			x					
22	database	Ward, P., Williams, A. M., & Bennett, S. J. (2002). Visual search and biological motion perception in tennis. Research Quarterly for Exercise and Sport, 73, 107-112. https://doi.org/10.1080/02701367.2002.10608997	---	2002	tennis	video	full-body movement						x		simulated strokes
23	database	Williams, A. M., Ward, P., Knowles, J. M., & Smeeton, N. J. (2002). Anticipation skill in a real-world task: Measurement, training, and transfer in tennis. Journal of Experimental Psychology: Applied, 8, 259-270. https://doi.org/10.1037/1076-898X.8.4.259	1	2002	tennis	video	full-body movement						x		simulated strokes
24	database	Farrow, D., & Abernethy, B. (2003). Do expertise and the degree of perception-action coupling affect natural anticipatory performance? Perception, 32(9), 1127-1139. https://doi.org/10.1068/p3323	1	2003	tennis	in-situ	verbal & full-body movement		x					x	both response mode conditions compared; with ball interception possible
25	database	Farrow, D., & Abernethy, B. (2003). Do expertise and the degree of perception-action coupling affect natural anticipatory performance? Perception, 32(9), 1127-1139. https://doi.org/10.1068/p3323	2	2003	tennis	in-situ	verbal & full-body movement		x					x	both response mode conditions compared; with ball interception possible
26	database	Crognier, L., & Féry, Y. A. (2005). Effect of tactical initiative on predicting passing shots in tennis. Applied Cognitive Psychology, 19(5), 637-649. https://doi.org/10.1002/Acp.1100	---	2005	tennis	in-situ	full-body movement							x	
27	database	Farrow, D., Abernethy, B., & Jackson, R. C. (2005). Probing expert anticipation with the temporal occlusion paradigm: Experimental investigations of some methodological issues. Motor Control, 9(3), 330-349. https://doi.org/10.1123/mcj.9.3.330	1	2005	tennis	video	paper-pen	x							
28	database	Farrow, D., Abernethy, B., & Jackson, R. C. (2005). Probing expert anticipation with the temporal occlusion paradigm: Experimental investigations of some methodological issues. Motor Control, 9(3), 330-349. https://doi.org/10.1123/mcj.9.3.330	2	2005	tennis	in-situ	full-body movement							x	with ball interception to the best of participants' ability
29	database	Shim, J., Carlton, L. G., Chow, J. W., & Chae, W. S. (2005). The use of anticipatory visual cues by highly	1	2005	tennis	video & in-situ	full-body movement						x		no ball interception due to use of liquid crystal goggles

no	retrieved via	reference	study	year	sport	stimulus presentation	response mode	level of perception-action coupling							note
								0	*1*	*2*	*3*	*4*	*5*	*6*	
		skilled tennis players. Journal of Motor Behavior, 37(2), 164-175. https://doi.org/10.3200/JMBR.37.2.164-175													
30	database	Shim, J., Carlton, L. G., Chow, J. W., & Chae, W. S. (2005). The use of anticipatory visual cues by highly skilled tennis players. Journal of Motor Behavior, 37(2), 164-175. https://doi.org/10.3200/JMBR.37.2.164-175	2	2005	tennis	in-situ	full-body movement							x	with ball interception
31	other method	Shim, J., Miller, G., & Lutz, R. (2005). Visual cues and information used to anticipate tennis ball shot and placement. Journal of Sport Behavior, 28(2), 186-200.	---	2005	tennis	video	full-body movement						x		simulated strokes
32	database	Hagemann, N., & Strauß, B. (2006). Perzeptive Expertise von Badmintonspielern [Perceptual expertise in badminton players]. Zeitschrift für Psychologie, 214(1), 37-47. https://doi.org/10.1026/0044-3409.214.1.37	---	2006	badminton	video	mouse click (1)	x							mouse click on a court representation (digital analogue to paper-pen responses)
33	database	Shim, J., Carlton, L. G., & Kwon, Y. H. (2006). Perception of kinematic characteristics of tennis strokes for anticipating stroke type and direction. Research Quarterly for Exercise and Sport, 77(3), 326-339. https://doi.org/10.1080/02701367.2006.10599367	2	2006	tennis	video	full-body movement						x		simulated strokes
34	database	Abernethy, B., & Zawi, K. (2007). Pickup of essential kinematics underpins expert perception of movement patterns. Journal of Motor Behavior, 39(5), 353-367. https://doi.org/10.3200/JMBR.39.5.353-368	1	2007	badminton	video	paper-pen	x							
35	database	Abernethy, B., & Zawi, K. (2007). Pickup of essential kinematics underpins expert perception of movement patterns. Journal of Motor Behavior, 39(5), 353-367. https://doi.org/10.3200/JMBR.39.5.353-368	2	2007	badminton	video	paper-pen	x							
36	database	Abernethy, B., & Zawi, K. (2007). Pickup of essential kinematics underpins expert perception of movement patterns. Journal of Motor Behavior, 39(5), 353-367. https://doi.org/10.3200/JMBR.39.5.353-368	3	2007	badminton	video	paper-pen	x							
37	database	Jackson, R. C., & Mogan, P. (2007). Advance visual information, awareness, and anticipation skill. Journal of Motor Behavior, 39(5), 341-351. https://doi.org/10.3200/JMBR.39.5.341-352	---	2007	tennis	video	full-body movement	(x)					x		additionally verbal expression of anticipated stroke outcome at about the same time as simulated stroke
38	database	Reina, R., Moreno, F. J., & Sanz, D. (2007). Visual behavior and motor responses of novice and experienced wheelchair tennis players relative to the service return. ADAPTED PHYSICAL ACTIVITY	---	2007	tennis	video & in-situ	simplified movement					x			no differentiation in PAC levels due to same response mode in different stimulus presentation conditions

								level of perception-action coupling							
**no**	**retrieved via**	**reference**	**study**	**year**	**sport**	**stimulus presentation**	**response mode**	*0*	*1*	*2*	*3*	*4*	*5*	*6*	**note**
		QUARTERLY, 24(3), 254-271. https://doi.org/10.1123/apaq.24.3.254													
39	database	Wright, M. J., & Jackson, R. C. (2007). Brain regions concerned with perceptual skills in tennis: An fMRI study. International Journal of Psychophysiology, 63(2), 214-220. https://doi.org/10.1016/j.ijpsycho.2006.03.018	---	2007	tennis	video	button press			x					
40	database	Abernethy, B., Zawi, K., & Jackson, R. C. (2008). Expertise and attunement to kinematic constraints. Perception, 37(6), 931-948. https://doi.org/10.1068/P5340	1	2008	badminton	video	paper-pen	x							
41	database	Abernethy, B., Zawi, K., & Jackson, R. C. (2008). Expertise and attunement to kinematic constraints. Perception, 37(6), 931-948. https://doi.org/10.1068/P5340	2	2008	badminton	video	paper-pen	x							
42	database	Abernethy, B., Zawi, K., & Jackson, R. C. (2008). Expertise and attunement to kinematic constraints. Perception, 37(6), 931-948. https://doi.org/10.1068/P5340	3	2008	badminton	video	paper-pen	x							
43	database	Huys, R., Smeeton, N. J., Hodges, N. J., Beek, P. J., & Williams, A. M. (2008). On the dynamic information underlying visual anticipation skill. Perception & Psychophysics, 70(7), 1217-1234. https://doi.org/10.3758/Pp.70.7.1217	2	2008	tennis	video	verbal		x						
44	database	Huys, R., Smeeton, N. J., Hodges, N. J., Beek, P. J., & Williams, A. M. (2008). On the dynamic information underlying visual anticipation skill. Perception & Psychophysics, 70(7), 1217-1234. https://doi.org/10.3758/Pp.70.7.1217	3	2008	tennis	video	verbal		x						
45	other method	Fukuhara, K., Ida, H., Kusubori, S., & Ishii, M. (2009). Anticipatory judgment of tennis serve: A comparison between video images and computer graphics animation. International Journal of Sport and Health Science, 7, 12-22.	1	2009	tennis	video	verbal		x						
46	other method	Fukuhara, K., Ida, H., Kusubori, S., & Ishii, M. (2009). Anticipatory judgment of tennis serve: A comparison between video images and computer graphics animation. International Journal of Sport and Health Science, 7, 12-22.	2	2009	tennis	video	verbal		x						
47	database	Hagemann, N. (2009). The advantage of being left-handed in interactive sports. Attention, Perception, & Psychophysics, 71(7), 1641-1648. https://doi.org/10.3758/App.71.7.1641	---	2009	tennis	video	mouse click (1)	x							mouse click on a court representation (digital analogue to paper-pen responses)

								level of perception-action coupling							
no	retrieved via	reference	study	year	sport	stimulus presentation	response mode	*0*	*1*	*2*	*3*	*4*	*5*	*6*	note
48	database	Huys, R., Cañal-Bruland, R., Hagemann, N., Beek, P. J., Smeeton, N. J., & Williams, A. M. (2009). Global information pickup underpins anticipation of tennis shot direction. Journal of Motor Behavior, 41(2), 158-170. https://doi.org/10.3200/jmbr.41.2.158-171	1	2009	tennis	video	paper-pen	x							
49	database	Huys, R., Cañal-Bruland, R., Hagemann, N., Beek, P. J., Smeeton, N. J., & Williams, A. M. (2009). Global information pickup underpins anticipation of tennis shot direction. Journal of Motor Behavior, 41(2), 158-170. https://doi.org/10.3200/jmbr.41.2.158-171	2	2009	tennis	video	paper-pen	x							
50	database	Jackson, R. C., Abernethy, B., & Wernhart, S. (2009). Sensitivity to fine-grained and coarse visual information: The effect of blurring on anticipation skill. International Journal of Sport Psychology, 40(4), 461-475.	---	2009	tennis	video	verbal		x						
51	database	Rowe, R. M., Horswill, M. S., Kronvall-Parkinson, M., Poulter, D. R., & McKenna, F. P. (2009). The effect of disguise on novice and expert tennis players' anticipation ability. Journal of Applied Sport Psychology, 21(2), 178-185. https://doi.org/10.1080/10413200902785811	---	2009	tennis	video	paper-pen	x							
52	database	Williams, A. M., Huys, R., Cañal-Bruland, R., & Hagemann, N. (2009). The dynamical information underpinning anticipation skill. Human Movement Science, 28(3), 362-370. https://doi.org/10.1016/j.humov.2008.10.006	---	2009	tennis	video	paper-pen	x							
53	database	Cañal-Bruland, R., & Williams, A. M. (2010). Recognizing and predicting movement effects: Identifying critical movement features. Experimental Psychology, 57(4), 320-326. https://doi.org/10.1027/1618-3169/a000038	---	2010	tennis	video	button press			x					
54	database	Wright, M. J., Bishop, D. T., Jackson, R. C., & Abernethy, B. (2010). Functional MRI reveals expert-novice differences during sport-related anticipation. Neuroreport, 21(2), 94-98. https://doi.org/10.1097/Wnr.0b013e328333dff2	1	2010	badminton	video	button press			x					
55	database	Wright, M. J., Bishop, D. T., Jackson, R. C., & Abernethy, B. (2010). Functional MRI reveals expert-novice differences during sport-related anticipation. Neuroreport, 21(2), 94-98. https://doi.org/10.1097/Wnr.0b013e328333dff2	2	2010	badminton	video	button press			x					
56	database	Cañal-Bruland, R., van Ginneken, W. F., van der Meer, B. R., & Williams, A. M. (2011). The effect of local kinematic changes on anticipation judgments.	---	2011	tennis	video	button press			x					

								level of perception-action coupling							
**no**	**retrieved via**	**reference**	**study**	**year**	**sport**	**stimulus presentation**	**response mode**	*0*	*1*	*2*	*3*	*4*	*5*	*6*	**note**
		Human Movement Science, 30(3), 495-503. https://doi.org/10.1016/j.humov.2010.10.001													
57	database	Ida, H., Fukuhara, K., Kusubori, S., & Ishii, M. (2011). A study of kinematic cues and anticipatory performance in tennis using computational manipulation and computer graphics. Behavior Research Methods, 43(3), 781-790. https://doi.org/10.3758/s13428-011-0084-x	---	2011	tennis	video	visual analogue scale	x							
58	database	Ida, H., Fukuhara, K., Sawada, M., & Ishii, M. (2011). Quantitative relation between server motion and receiver anticipation in tennis: Implications of responses to computer-simulated motions. Perception, 40(10), 1221-1236. https://doi.org/10.1068/p7041	---	2011	tennis	video	visual analogue scale	x							
59	database	Jin, H., Xu, G., Zhang, J. X., Gao, H., Ye, Z., Wang, P., Lin, H., Mo, L., & Lin, C. D. (2011). Event-related potential effects of superior action anticipation in professional badminton players. Neuroscience Letters, 492(3), 139-144. https://doi.org/10.1016/j.neulet.2011.01.074	---	2011	badminton	video	button press			x					
60	database	Loffing, F., Wilkes, T., & Hagemann, N. (2011). Skill level and graphical detail shape perceptual judgments in tennis. Perception, 40(12), 1447-1456. https://doi.org/10.1068/p7035	---	2011	tennis	video	button press			x					
61	database	Mecheri, S., Gillet, E., Thouvarecq, R., & Leroy, D. (2011). Are visual cue masking and removal techniques equivalent for studying perceptual skills in sport?. Perception, 40(4), 474-489. https://doi.org/10.1068/p6828	---	2011	tennis	video	button press			x					
62	database	Smeeton, N. J., & Huys, R. (2011). Anticipation of tennis-shot direction from whole-body movement: The role of movement amplitude and dynamics. Human Movement Science, 30(5), 957-965. https://doi.org/10.1016/j.humov.2010.07.012	---	2011	tennis	video	paper-pen	x							
63	database	Wright, M. J., Bishop, D. T., Jackson, R. C., & Abernethy, B. (2011). Cortical fMRI activation to opponents' body kinematics in sport-related anticipation: Expert-novice differences with normal and point-light video. Neuroscience Letters, 500(3), 216-221. https://doi.org/10.1016/j.neulet.2011.06.045	---	2011	badminton	video	button press			x					
64	database	Farrow, D., & Reid, M. (2012). The contribution of situational probability information to anticipatory skill. Journal of Science and Medicine in Sport, 15(4), 368-373. https://doi.org/10.1016/j.jsams.2011.12.007	---	2012	tennis	video	finger tip			x					

								level of perception-action coupling							
no	retrieved via	reference	study	year	sport	stimulus presentation	response mode	*0*	*1*	*2*	*3*	*4*	*5*	*6*	note
65	database	Ida, H., Fukuhara, K., Ishii, M., & Inoue, T. (2013). Perceptual response and information pick-up strategies within a family of sports. Human Movement Science, 32(1), 106-120. https://doi.org/10.1016/j.humov.2012.08.002	---	2013	tennis	video	visual analogue scale	x							
66	database	Alder, D., Ford, P. R., Causer, J., & Williams, A. M. (2014). The coupling between gaze behavior and opponent kinematics during anticipation of badminton shots. Human Movement Science, 37(0), 167-179. https://doi.org/10.1016/j.humov.2014.07.002	---	2014	badminton	video	full-body movement	(x)					x		additionally verbal expression of anticipated stroke outcome at about the same time as simulated stroke
67	database	Balser, N., Lorey, B., Pilgramm, S., Naumann, T., Kindermann, S., Stark, R., Zentgraf, K., Williams, A. M., & Munzert, J. (2014). The influence of expertise on brain activation of the Action Observation Network during anticipation of tennis and volleyball serves [Original Research]. Frontiers in Human Neuroscience, 8. https://doi.org/10.3389/fnhum.2014.00568	---	2014	tennis	video	button press			x					volleyball serves were also shown
68	database	Balser, N., Lorey, B., Pilgramm, S., Stark, R., Bischoff, M., Zentgraf, K., Williams, A. M., & Munzert, J. (2014). Prediction of human actions: Expertise and task-related effects on neural activation of the action observation network. Human Brain Mapping, 35(8), 4016-4034. https://doi.org/10.1002/hbm.22455	---	2014	tennis	video	button press			x					
69	database	Bischoff, M., Zentgraf, K., Pilgramm, S., Stark, R., Krüger, B., & Munzert, J. (2014). Anticipating action effects recruits audiovisual movement representations in the ventral premotor cortex. Brain and Cognition, 92(0), 39-47. https://doi.org/10.1016/j.bandc.2014.09.010	---	2014	table tennis	video	button press			x					
70	database	Loffing, F., & Hagemann, N. (2014). On-court position influences skilled tennis players' anticipation of shot outcome. Journal of Sport & Exercise Psychology, 36(1), 14-26. https://doi.org/10.1123/jsep.2013-0082	---	2014	tennis	video	button press			x					
71	database	Park, S. H., Kim, S., Kwon, M., & Christou, E. A. (2015). Differential contribution of visual and auditory information to accurately predict the direction and rotational motion of a visual stimulus. Applied Physiology, Nutrition and Metabolism, 41(3), 244-248. https://doi.org/10.1139/apnm-2015-0390	---	2015	table tennis	video	button press			x					
72	database	Cocks, A. J., Jackson, R. C., Bishop, D. T., & Williams, A. M. (2016). Anxiety, anticipation and	---	2016	tennis	video	simplified movement					x			

								level of perception-action coupling							
**no**	**retrieved via**	**reference**	**study**	**year**	**sport**	**stimulus presentation**	**response mode**	*0*	*1*	*2*	*3*	*4*	*5*	*6*	**note**
		contextual information: A test of attentional control theory. Cognition and Emotion, 30(6), 1037-1048. https://doi.org/10.1080/02699931.2015.1044424													
73	database	Loffing, F., Sölter, F., Hagemann, N., & Strauss, B. (2016). On-court position and handedness in visual anticipation of stroke direction in tennis. Psychology of Sport and Exercise, 27, 195-204. https://doi.org/10.1016/j.psychsport.2016.08.014	---	2016	tennis	video	button press			x					
74	database	Murphy, C. P., Jackson, R. C., Cooke, K., Roca, A., Benguigui, N., & Williams, A. M. (2016). Contextual information and perceptual-cognitive expertise in a dynamic, temporally-constrained task. Journal of Experimental Psychology: Applied, 22(4), 455-470. https://doi.org/10.1037/xap0000094	1	2016	tennis	video	full-body movement	(x)					x		additionally verbal expression of anticipated stroke outcome at about the same time as simulated stroke
75	database	Murphy, C. P., Jackson, R. C., Cooke, K., Roca, A., Benguigui, N., & Williams, A. M. (2016). Contextual information and perceptual-cognitive expertise in a dynamic, temporally-constrained task. Journal of Experimental Psychology: Applied, 22(4), 455-470. https://doi.org/10.1037/xap0000094	2	2016	tennis	video	full-body movement	(x)					x		additionally verbal expression of anticipated stroke outcome at about the same time as simulated stroke
76	database	Piras, A., Lanzoni, I. M., Raffi, M., Persiani, M., & Squatrito, S. (2016). The within-task criterion to determine successful and unsuccessful table tennis players. International Journal of Sports Science and Coaching, 11(4), 523-531. https://doi.org/10.1177/1747954116655050	---	2016	table tennis	video	button press			x					
77	database	Xu, H., Wang, P., Ye, Z., Di, X., Xu, G., Mo, L., Lin, H., Rao, H., & Jin, H. (2016). The role of medial frontal cortex in action anticipation in professional badminton players. Frontiers in Psychology, 7(NOV), Article 1817. https://doi.org/10.3389/fpsyg.2016.01817	---	2016	badminton	video	button press			x					
78	database	Denis, D., Rowe, R., Williams, A. M., & Milne, E. (2017). The role of cortical sensorimotor oscillations in action anticipation. NeuroImage, 146, 1102-1114. https://doi.org/10.1016/j.neuroimage.2016.10.022	---	2017	tennis	video	button press			x					
79	database	Fukuhara, K., Ida, H., Ogata, T., Ishii, M., & Higuchi, T. (2017). The role of proximal body information on anticipatory judgment in tennis using graphical information richness. PLoS ONE, 12(7), e0180985. https://doi.org/10.1371/journal.pone.0180985	---	2017	tennis	video	mouse click (2)			x					mouse click (left/right) as directional response similar to button press
80	database	Liu, S., Ritchie, J., Sáenz-Moncaleano, C., Ward, S. K., Paulsen, C., Klein, T., Gutierrez, O., &	---	2017	tennis	video	button press			x					

								level of perception-action coupling							
no	retrieved via	reference	study	year	sport	stimulus presentation	response mode	*0*	*1*	*2*	*3*	*4*	*5*	*6*	note
		Tenenbaum, G. (2017). 3D technology of Sony Bloggie has no advantage in decision-making of tennis serve direction: A randomized placebo-controlled study. European Journal of Sport Science, 17(5), 603-610. https://doi.org/10.1080/17461391.2017.1301561													
81	database	Alder, D. B., Ford, P. R., Causer, J., & Williams, A. M. (2018). The effect of anxiety on anticipation, allocation of attentional resources, and visual search behaviours. Human Movement Science, 61, 81-89. https://doi.org/10.1016/j.humov.2018.07.002	---	2018	badminton	video	full-body movement						x		simulated strokes
82	database	Cañal-Bruland, R., Müller, F., Lach, B., & Spence, C. (2018). Auditory contributions to visual anticipation in tennis. Psychology of Sport and Exercise, 36, 100-103. https://doi.org/10.1016/j.psychsport.2018.02.001	---	2018	tennis	video	mouse click (1)	x							mouse click on a court representation (digital analogue to paper-pen responses)
83	database	Fukuhara, K., Maruyama, T., Ida, H., Ogata, T., Sato, B., Ishii, M., & Higuchi, T. (2018). Can slow-motion footage of forehand strokes be used to immediately improve anticipatory judgments in tennis?. Frontiers in Psychology, 9. https://doi.org/10.3389/fpsyg.2018.01830	---	2018	tennis	video	mouse click (2)			x					mouse click (left/right) as directional response similar to button press
84	database	Jalali, S., Martin, S. E., Murphy, C. P., Solomon, J. A., & Yarrow, K. (2018). Classification Videos Reveal the Visual Information Driving Complex Real-World Speeded Decisions. Frontiers in Psychology, 9. https://doi.org/10.3389/fpsyg.2018.02229	---	2018	tennis	video	simplified movement					x			
85	database	Murphy, C. P., Jackson, R. C., & Williams, A. M. (2018). The role of contextual information during skilled anticipation. Quarterly Journal of Experimental Psychology, 71(10), 2070-2087. https://doi.org/10.1177/1747021817739201	1	2018	tennis	video	full-body movement	(x)					x		additionally verbal expression of anticipated stroke outcome at about the same time as simulated stroke
86	database	Murphy, C. P., Jackson, R. C., & Williams, A. M. (2018). The role of contextual information during skilled anticipation. Quarterly Journal of Experimental Psychology, 71(10), 2070-2087. https://doi.org/10.1177/1747021817739201	2	2018	tennis	video	full-body movement	(x)					x		additionally verbal expression of anticipated stroke outcome at about the same time as simulated stroke
87	database	Shangguan, R., & Che, Y. (2018). The difference in perceptual anticipation between professional tennis athletes and second-grade athletes before batting. Frontiers in Psychology, 9(AUG), Article 1541. https://doi.org/10.3389/fpsyg.2018.01541	---	2018	tennis	image	button press	x							
88	database	Zhao, Q., Lu, Y., Jaquess, K. J., & Zhou, C. (2018). Utilization of cues in action anticipation in table	---	2018	table tennis	video	button press			x					

								level of perception-action coupling							
**no**	**retrieved via**	**reference**	**study**	**year**	**sport**	**stimulus presentation**	**response mode**	*0*	*1*	*2*	*3*	*4*	*5*	*6*	**note**
		tennis players. Journal of Sports Sciences, 36(23), 2699-2705. https://doi.org/10.1080/02640414.2018.1462545													
89	database	Alder, D. B., Broadbent, D. P., Stead, J., & Poolton, J. (2019). The impact of physiological load on anticipation skills in badminton: From testing to training. Journal of Sports Sciences, 37(16), 1816-1823. https://doi.org/10.1080/02640414.2019.1596051	1	2019	badminton	video	full-body movement						x		simulated strokes
90	database	Huesmann, K., & Loffing, F. (2019). Contextual cue utilization in visual anticipation in tennis: On the role of an opponent's on-court position and skill [journal article]. German Journal of Exercise and Sport Research, 49(3), 304-312. https://doi.org/10.1007/s12662-019-00597-y	---	2019	tennis	video	button press			x					
91	database	Ida, H., Fukuhara, K., Ishii, M., & Inoue, T. (2019). Anticipatory judgements associated with vision of an opponent's end-effector: An approach by motion perturbation and spatial occlusion. Quarterly Journal of Experimental Psychology, 72(5), 1131-1140. https://doi.org/10.1177/1747021818782419	---	2019	tennis	video	visual analogue scale	x							
92	database	Jalali, S., Martin, S. E., Ghose, T., Buscombe, R. M., Solomon, J. A., & Yarrow, K. (2019). Information accrual from the period preceding racket-ball contact for tennis ground strokes: Inferences from stochastic masking. Frontiers in Psychology, 10(AUG), Article 1969. https://doi.org/10.3389/fpsyg.2019.01969	---	2019	tennis	video	simplified movement					x			
93	database	Müller, F., Jauernig, L., & Cañal-Bruland, R. (2019). The sound of speed: How grunting affects opponents' anticipation in tennis. PLoS ONE, 14(4), e0214819. https://doi.org/10.1371/journal.pone.0214819	---	2019	tennis	video	mouse click (1)	x							mouse click on a court representation (digital analogue to paper-pen responses)
94	database	Murphy, C. P., Jackson, R. C., & Williams, A. M. (2019). Informational constraints, option generation, and anticipation. Psychology of Sport and Exercise, 41, 54-62. https://doi.org/10.1016/j.psychsport.2018.11.012	---	2019	tennis	video	paper-pen	x							
95	database	Park, S. H., Ryu, D., Uiga, L., Masters, R., Abernethy, B., & Mann, D. L. (2019). Falling for a Fake: The Role of Kinematic and Non-kinematic Information in Deception Detection. Perception, 48(4), 330-337. https://doi.org/10.1177/0301006619837874	---	2019	badminton	video	button press			x					

no	retrieved via	reference	study	year	sport	stimulus presentation	response mode	level of perception-action coupling							note
								0	*1*	*2*	*3*	*4*	*5*	*6*	
96	database	Roberts, J. W., Keen, B., & Kawycz, S. (2019). Anticipation of badminton serves during naturalistic match-play: a case for the post-performance analysis of perceptual-cognitive skills. Journal of Sports Medicine and Physical Fitness, 59(12), 1951-1955. https://doi.org/10.23736/S0022-4707.19.09540-9	---	2019	badminton	in-situ	full-body movement							x	with ball interception
97	database	Wang, Y. Y., Lu, Y. Z., Deng, Y. Q., Gu, N., Tiina, P., & Zhou, C. L. (2019). Predicting domain-specific actions in expert table tennis players activates the semantic brain network. NeuroImage, 200, 482-489. https://doi.org/10.1016/j.neuroimage.2019.06.035	---	2019	table tennis	video	button press			x					
98	database	Lu, Y., Yang, T., Hatfield, B. D., Cong, F., & Zhou, C. (2020). Influence of cognitive-motor expertise on brain dynamics of anticipatory-based outcome processing. Psychophysiology, 57(2), e13477. https://doi.org/https://doi.org/10.1111/psyp.13477	---	2020	table tennis	video	button press			x					
99	database	Cañal-Bruland, R., Meyerhoff, H. S., & Müller, F. (2022). Context modulates the impact of auditory information on visual anticipation. Cognitive Research: Principles and Implications, 7(1), 76. https://doi.org/10.1186/s41235-022-00425-2	1	2022	tennis	video	mouse click (1)	x							mouse click on a court representation (digital analogue to paper-pen responses)
100	database	Cañal-Bruland, R., Meyerhoff, H. S., & Müller, F. (2022). Context modulates the impact of auditory information on visual anticipation. Cognitive Research: Principles and Implications, 7(1), 76. https://doi.org/10.1186/s41235-022-00425-2	2	2022	tennis	video	mouse click (1)	x							mouse click on a court representation (digital analogue to paper-pen responses)
101	database	Navia, J. A., Avilés, C., Dicks, M., & Ruiz-Pérez, L. M. (2022). The spatiotemporal control of expert tennis players when returning first serves: A perception-action perspective. Journal of Sports Sciences, 40(1), 16-23. https://doi.org/10.1080/02640414.2021.1976484	---	2022	tennis	in-situ	full-body movement							x	with ball interception
102	database	Ren, P., Song, T., Chi, L., Wang, X., & Miao, X. (2022). The Adverse Effect of Anxiety on Dynamic Anticipation Performance [Original Research]. Frontiers in Psychology, 13. https://doi.org/10.3389/fpsyg.2022.823989	---	2022	table tennis	video	button press			x					
103	database	Robertson, K., De Waelle, S., Deconinck, F. J., & Lenoir, M. (2022). Differences in expertise level for anticipatory skill between badminton ‘in game’ strokes and serves. International Journal of Sports Science & Coaching, 17(4), 782-791. https://doi.org/10.1177/17479541211046910	---	2022	badminton	video	full-body movement						x		simulated strokes

								level of perception-action coupling							
**no**	**retrieved via**	**reference**	**study**	**year**	**sport**	**stimulus presentation**	**response mode**	*0*	*1*	*2*	*3*	*4*	*5*	*6*	**note**
104	database	Shangguan, R., Tang, J. L., & Che, Y. Y. (2022). The influence of emotional state on perceptual anticipation of tennis players in Stalemate Stage. International Journal of Sport Psychology, 53(3), 267-280. https://doi.org/10.7352/IJSP.2021.52.267	---	2022	tennis	video	button press			x					
105	database	Wang, Y., Ji, Q., Fu, R., Zhang, G., Lu, Y., & Zhou, C. (2022). Hand-related action words impair action anticipation in expert table tennis players: Behavioral and neural evidence. Psychophysiology, 59(1), e13942. https://doi.org/10.1111/psyp.13942	1	2022	table tennis	video	button press			x					
106	database	Wang, Y., Ji, Q., Fu, R., Zhang, G., Lu, Y., & Zhou, C. (2022). Hand-related action words impair action anticipation in expert table tennis players: Behavioral and neural evidence. Psychophysiology, 59(1), e13942. https://doi.org/10.1111/psyp.13942	2	2022	table tennis	video	button press			x					
107	database	Costa, S., Berchicci, M., Bianco, V., Croce, P., Di Russo, F., Quinzi, F., Bertollo, M., & Zappasodi, F. (2023). Brain dynamics of visual anticipation during spatial occlusion tasks in expert tennis players. Psychology of Sport and Exercise, 65, Article 102335. https://doi.org/10.1016/j.psychsport.2022.102335	---	2023	tennis	image	button press	x							
108	database	Dai, C., Peng, Z., Wang, L., Song, T., Xu, L., Xu, M., & Shao, Y. (2023). Total sleep deprivation reduces the table tennis anticipation performance of young men: A functional magnetic resonance imaging study. iScience, 26(10), Article 107973. https://doi.org/10.1016/j.isci.2023.107973	---	2023	table tennis	video	button press			x					
109	database	De Waelle, S., Robertson, K., Deconinck, F. J. A., & Lenoir, M. (2023). The Use of Contextual Information for Anticipation of Badminton Shots in Different Expertise Levels. Research Quarterly for Exercise and Sport, 94(1), 15-23. https://doi.org/10.1080/02701367.2021.1934378	---	2023	badminton	video	full-body movement						x		simulated strokes
110	database	Fujita, R. A., Santos, D. P. R., Barbosa, R. N., Vieira, L. H. P., Santiago, P. R. P., Zagatto, A. M., & Gomes, M. M. (2023). Auditory Information Reduces Response Time for Ball Rotation Perception, Increasing Counterattack Performance in Table Tennis. Research Quarterly for Exercise and Sport, 94(1), 55-63. https://doi.org/10.1080/02701367.2021.1939252	1	2023	table tennis	video	button press			x					
111	database	Fujita, R. A., Santos, D. P. R., Barbosa, R. N., Vieira, L. H. P., Santiago, P. R. P., Zagatto, A. M., & Gomes,	2	2023	table tennis	in-situ	full-body movement							x	with ball interception

no	retrieved via	reference	study	year	sport	stimulus presentation	response mode	level of perception-action coupling							note
								0	*1*	*2*	*3*	*4*	*5*	*6*	
		M. M. (2023). Auditory Information Reduces Response Time for Ball Rotation Perception, Increasing Counterattack Performance in Table Tennis. Research Quarterly for Exercise and Sport, 94(1), 55-63. https://doi.org/10.1080/02701367.2021.1939252													
112	database	Müller, F., Will, J. J. R., & Cañal-Bruland, R. (2023). Multisensory integration in anticipation: moderating effects of time and task constraints. International Journal of Sport and Exercise Psychology. https://doi.org/10.1080/1612197X.2023.2224821	1	2023	tennis	video	finger swipe				x				
113	database	Müller, F., Will, J. J. R., & Cañal-Bruland, R. (2023). Multisensory integration in anticipation: moderating effects of time and task constraints. International Journal of Sport and Exercise Psychology. https://doi.org/10.1080/1612197X.2023.2224821	2	2023	tennis	video	finger tip			x					
114	database	Wang, X., Ren, P., Miao, X., Zhang, X., Qian, Y., & Chi, L. (2023). Attention Load Regulates the Facilitation of Audio-Visual Information on Landing Perception in Badminton. Perceptual and Motor Skills, 130(4), 1687-1713. https://doi.org/10.1177/00315125231180893	1	2023	badminton	video	button press			x					
115	database	Wang, X., Ren, P., Miao, X., Zhang, X., Qian, Y., & Chi, L. (2023). Attention Load Regulates the Facilitation of Audio-Visual Information on Landing Perception in Badminton. Perceptual and Motor Skills, 130(4), 1687-1713. https://doi.org/10.1177/00315125231180893	2	2023	badminton	video	button press			x					
